# Anti-Inflammatory Effects of *Alnus Sibirica* Extract on In Vitro and In Vivo Models

**DOI:** 10.3390/molecules25061418

**Published:** 2020-03-20

**Authors:** Jeongyoon Choi, Sunghee Moon, Hyemi Bae, Young-Won Kim, Yelim Seo, Hye Soo Wang, Min Won Lee, Hae Young Yoo, Jung-Ha Kim, Jae-Hong Ko, Inja Lim, Hyoweon Bang

**Affiliations:** 1 Department of Physiology, College of Medicine, Chung-Ang University, Seoul 06974, Korea; cju415@cau.ac.kr (J.C.); soghe@hanmail.net (S.M.); hyemiworld@cau.ac.kr (H.B.); stream00@nate.com (Y.-W.K.); dodqjf6@naver.com (Y.S.); akdongyi01@cau.ac.kr (J.-H.K.); injalim@naver.com (I.L.); 2 Laboratory of Pharmacognosy and Natural Product based Medicine, College of Pharmacy, Chung-Ang University, Seoul 06974, Korea; envi2308@hanmail.net (H.S.W.); mwlee@cau.ac.kr (M.W.L.); 3 Red Cross College of Nursing, Chung-Ang University, Seoul 06974, Korea; hyoo@cau.ac.kr; 4 Department of Family Medicine, College of Medicine, Chung-Ang University Hospital, 102 Heukseok-ro, Seoul 06973, Korea; girlpower219@cau.ac.kr

**Keywords:** *Alnus sibirica*, skin inflammation, house dust mite ointment (HDM), human dermal fibroblasts (HDFs), inflammatory stimulants, NC/Nga mice

## Abstract

*Alnus sibirica* extracts (ASex) have long been used in Oriental medicine to treat various conditions. To provide a scientific basis for this application and the underlying mechanism, we investigated the anti-inflammatory effects of ASex in vitro and in vivo. The in vitro model was established using human dermal fibroblasts (HDFs) treated with inflammatory stimulants (lipopolysaccharide, tumor necrosis factor-alpha, interferon-gamma). Lactate dehydrogenase and reverse transcription-polymerase chain reaction showed that ASex inhibited the increased expression of acute-phase inflammatory cytokines. The in vivo model was established by inducing skin inflammation in NC/Nga mice via the repeated application of house dust mite (HDM) ointment to the ears and back of the mice for eight weeks. HDM application increased the severity of skin lesions, eosinophil/mast cell infiltration, and serum immunoglobulin E levels, which were all significantly decreased by ASex treatment, demonstrating the same degree of protection as hydrocortisone. Overall, ASex showed excellent anti-inflammatory effects both in vitro and in vivo, suggesting its potential as an excellent candidate drug to reduce skin inflammation.

## 1. Introduction

Skin inflammation induces alterations to the stratum corneum, triggering the activation of immune cells, thereby increasing the production of immunoglobulin E (IgE)-dependent histamine releasing factor and IgE, and the antibody response results in substantial eosinophil infiltration [1]. The infiltration of various immune cells in turn induces the proliferation of other immune cells by the secretion of various cytokines along with the enhanced production of factors involved in skin formation/proliferation. In this regard, the activation of the NF-kB (nuclear factor kappa-light-chain-enhancer of activated B cells) transcription factor is essential to trigger the inflammatory response and the increase in mRNA and circulating levels of inflammatory cytokines, such as TNF-α [2]. The acute phase of skin inflammation involves the excessive production of the alarmin cytokines such as interleukin (IL)-33; non-specific inflammatory cytokines such as IL-1β, IL-8, and tumor necrosis factor-alpha (TNF-α); and acute inflammatory cytokines such as IL-4, IL-5, IL-6, IL-10, and IL-13 [3,4,5]. The chronic phase of skin inflammation involves only the inflammatory cytokines, including IL-12, IL-18, and interferon-gamma (IFN-γ).

The current treatment strategy for skin inflammation involves the suppression of histamine secretion and the alleviation of symptoms and signs with corticosteroids, antihistamines, antibiotics, cyclosporine, and interferons. Nevertheless, only a few drugs have been confirmed to have therapeutic effects, and their use is limited by side effects. Among these, corticosteroids remain the most commonly used drugs, despite side effects such as thinning/atrophy of the skin and delayed growth in pediatric patients [6,7]. Thus, the development of safe drugs without toxicity and side effects is very important. Natural products have relatively few serious side effects; however, it has been a challenge to identify natural products with a clear anti-inflammatory effect.

*Alnus sibirica* is a deciduous tree of the Betulaceae genus *Alnus*, which has long been used in Oriental medicine for the treatment of fever, bleeding, diarrhea, gastrointestinal disorders, lymphatic system diseases, and cancers [8]. *Alnus* species produce various compounds such as diarylheptanoids [9,10], flavonoids [11], tannins, and triterpenoids [12] with antioxidant, anti-inflammatory [13], and anti-atopic dermatitis (AD) activities [14] and demonstrate toxic properties to tumor cells [15]. Local application of *Alnus japonica* extract (AJex) to NC/Nga mice with AD-like skin lesions reduced the overexpression of Th2 cytokines, eosinophil infiltration, and serum IgE levels [16]. The intraperitoneal injection of oregonin isolated from AJex to NC/Nga mice also reduced inflammation-related markers [17]. Treatment with another compound of *Alnus* extract, hirsutenone, inhibited inflammation in keratinocytes induced by reactive oxygen species, suggesting its potentially beneficial effects in the treatment of inflammation at the cellular level [18]. Oral administration of fermented *A. sibirica* extract (ASex) to BALB/c mice reduced serum IgE levels and the expression levels of IL-4 and IFN-γ in splenocytes [19]. However, this study only showed the mitigating effects of ASex on systemic inflammation and did not address its potential effects on skin inflammation.

Recently, fibroblasts have been recognized to play extended roles beyond their main function in tissue repair as an important immune cell type, with greater effects than macrophages and keratinocytes during the transition from acute to chronic inflammation [3,20,21,22]. Fibroblasts mediate immune responses through the secretion of cytokines, chemokines, and growth factors. Choi et al. [3] reported that various inflammatory cytokines in human dermal fibroblasts (HDFs) were upregulated following treatment with inflammatory stimulants. In particular, increased expression levels of IL-33 induced by IFN-γ indicated a positive feedback loop in inflammation, which was suggested to be an essential mechanism for the transition of inflammation from the acute to chronic state.

Various animal models have been used to study skin inflammation to date. However, no animal model fully mimics the pathophysiology of a particular human disease. NC/Nga mice have long been adopted as an AD-like skin inflammation model, which are highly enriched in epidermal T cells and are thus useful for investigating human-like immune responses [23]. NC/Nga mice treated with house dust mite (HDM) ointment exhibit phenotypes similar to those in human AD, such as immunological changes and skin barrier damage [24,25].

**Figure 1:**
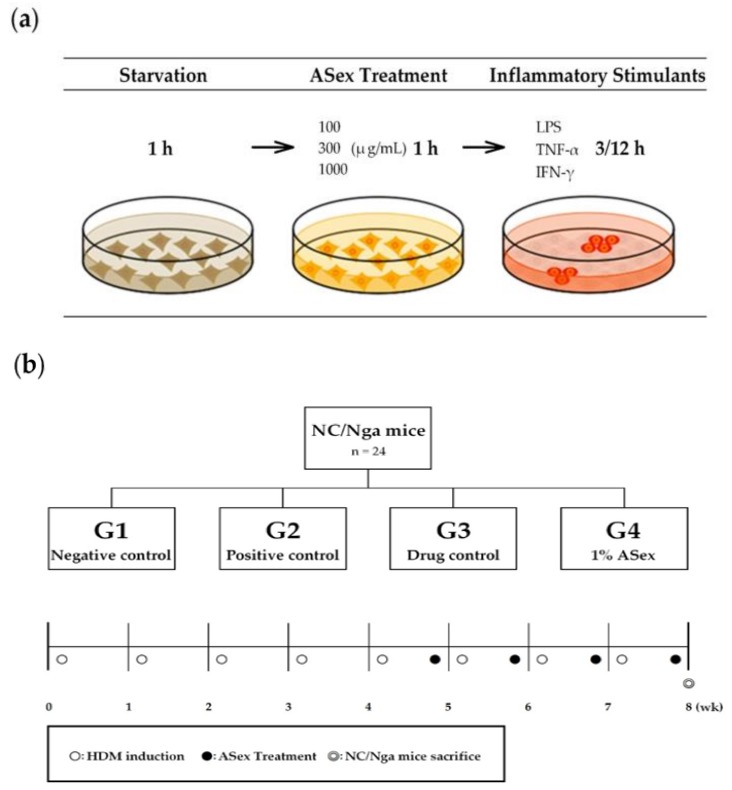
Overview of the in vitro and in vivo experiments. (**a**) The three steps for the induction of an inflammatory condition on cells; (**b**) Group names and descriptions for the 24 NC/Nga mice and schedules for the induction of inflammatory skin lesions on the skin and/or ears. LPS: lipopolysaccharide. TNF-α: tumor necrosis factor-alpha. IFN-γ: interferon-gamma. h: hour. G: group. HDM: house dust mite ointment. ASex: *Alnus sibirica* extract. Wk: week.

Therefore, to further evaluate the potential of ASex in attenuating skin inflammation and the underlying mechanism, in the present study, we induce skin inflammation using in vitro and in vivo models. In vitro experiments are performed to investigate the effects of ASex on cytokine expression induced by inflammatory stimulants in HDFs, whereas the in vivo experiment involves HDM-treated NC/Nga mice to examine the effects of ASex on immunological responses and skin lesions. A schematic of the experimental designs is shown in Figure 1. These results can provide a scientific basis for ASex as a new candidate natural drug in the treatment of skin inflammation.

## 2. Results

### 2.1. ASex Component Analysis

#### High-Performance Liquid Chromatography (HPLC)

**Figure 2:**
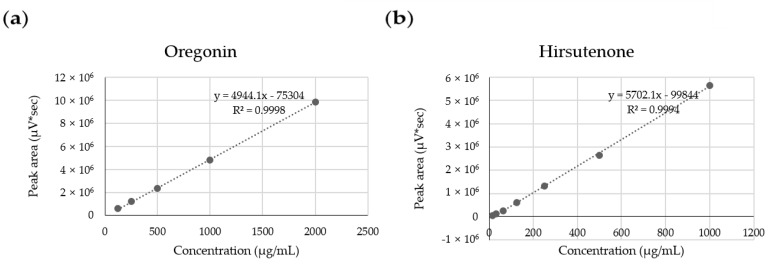
Linearity and correlation coefficients of effective (indicator) components (R^2^). (**a**) Oregonin; (**b**) hirsutenone.

**Figure 3:**
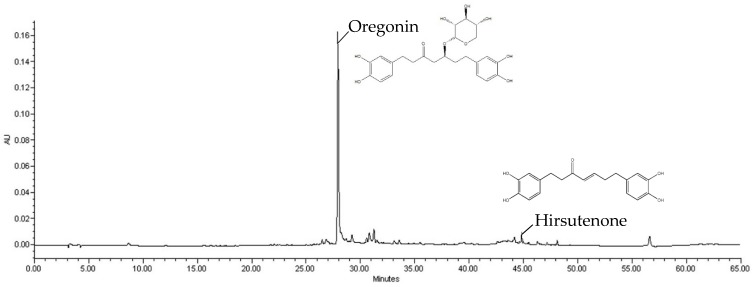
High-performance liquid chromatography (HPLC) result of *Alnus sibirica* extract (ASex) and its major compounds.

Several types of ASex were previously shown to significantly reduce the expression of inflammatory cytokines induced by inflammatory stimulants, although there were some differences depending on the collection area, tree parts (bark and leave), and extraction method [3]. Therefore, to select the extract for this study, ASex was prepared from the bark of *A. sibirica* collected from various regions in Korea, and the main components of each extract were evaluated by HPLC (Table 1). The linear regression equation (*y = ax + b, y = peak area, x*: sample concentration, *a*: slope of straight line, *b*: *y* intercept) was obtained by analyzing the content of surface components. Figure 2 demonstrates high linearity of the calibration curve with *R*^2^ > 0.999, confirming its suitability to evaluate the content of the indicator components. Oregonin and hirsutenone were identified as the main components in ASex from all regions. Since the yield of ASex and the oregonin content of the bark from the Jacheon area were relatively high (Table 1, Figure 3), this bark was selected for the experiment because it is more easily accessible, allowing for collection in large quantities and showing good extraction efficiency.

### 2.2. Inflammatory Cytokine Expression in HDFs: In Vitro Model

#### 2.2.1. Cytotoxicity: Lactate Dehydrogenase (LDH) Assay

**Figure 4:**
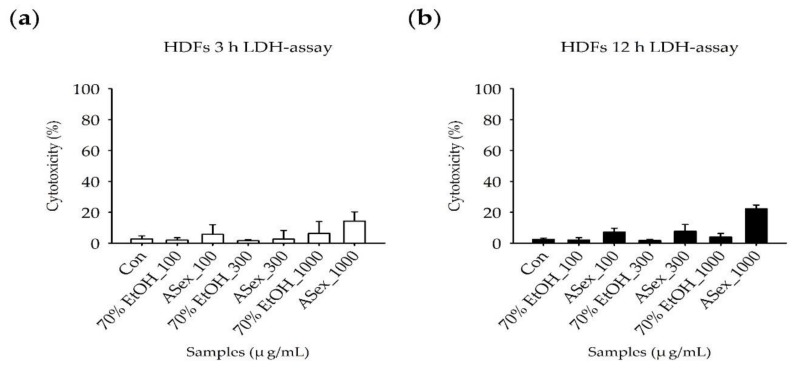
Cytotoxicity of ASex in human dermal fibroblasts (HDFs). (**a**) Treatment of HDFs with ASex for 3 h; (**b**) Treatment of HDFs with ASex for 12 h. ASex was applied to the cells at 100, 300, and 1000 μg/mL for the indicated periods before the LDH assay was performed. The results are expressed as the mean ± SD (standard deviation) of triplicate experiments (*n* = 3).

The LDH assay is a widely used method of determining the cytotoxicity of a new compound: if a compound shows higher cytotoxicity (40% or more) compared to the control sample, then it might be considered toxic to cells. According to the experimental protocol (Figure 1a) requiring LPS-stimulated HDFs to be incubated with TNF-α and IFN-γ for 3 h and 12 h, respectively, the LDH assay was performed to evaluate the cytotoxicity of ASex exposure for 3 h and 12 h. A vehicle control was added to confirm the effect of the 70% ethanol solution used to dissolve the sample. Less than 20% cytotoxicity was observed after treatment with ASex for 3 h, demonstrating that ASex does not have a cytotoxic effect, even at high concentration, and did not induce cell death (Figure 4a). The 12 h treatment of ASex was not inducing a significant cytotoxicity, but a slight cell membrane damage compared to each controls (Figure 4b).

#### 2.2.2. Expression of Inflammatory Cytokine Genes Induced by Inflammatory Stimulants in HDFs

**Figure 5:**
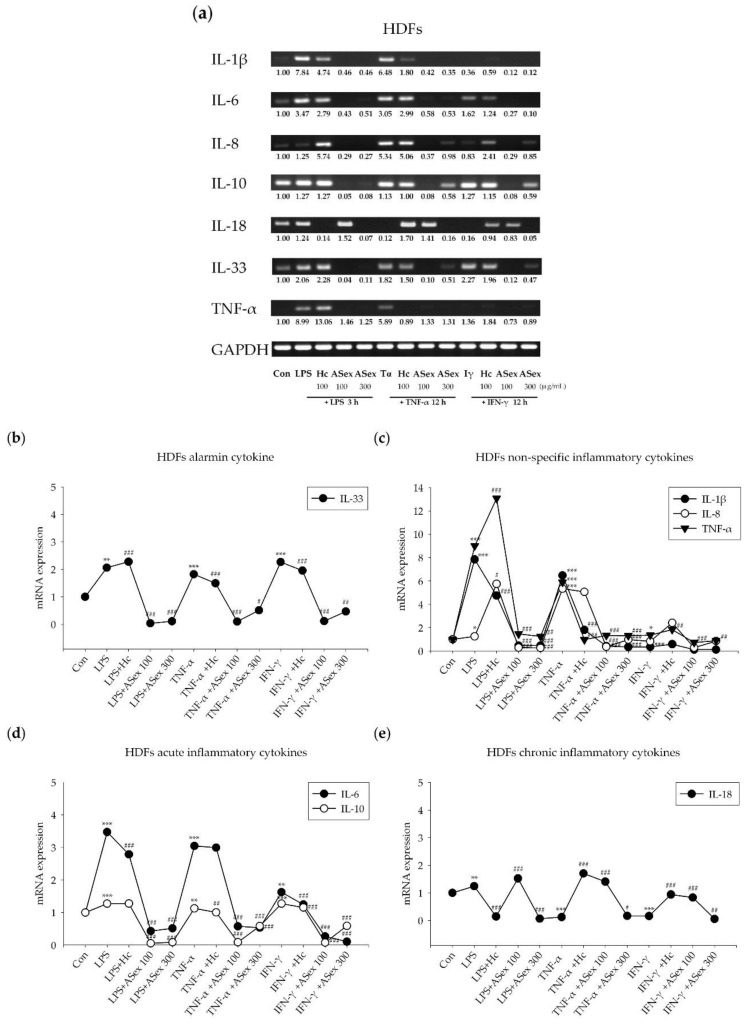
Changes in the mRNA expression levels of inflammatory cytokines in human dermal fibroblasts (HDFs) induced by inflammatory stimulants with or without ASex. (**a**): All of the inflammatory cytokines mRNA expression in HDFs; (**b**): Level of alarmin cytokine; (**c**): level of non-specific inflammatory cytokines; (**d**): level of acute inflammatory cytokines; (**e**): level of chronic inflammatory cytokines. The cells were cultured in starvation medium and treated with 100 and 300 μg/mL ASex for 1 h before treatment of the inflammatory stimulants as follows: 100 ng/mL LPS for 3 h, and 10 ng/mL TNF-α or IFN-γ for 12 h. The results are expressed as the mean ± SD of triplicate experiments (*n* = 3). Statistical significance was assessed by Student’s *t*-test and ANOVA: **p < 0.01, ***p < 0.001 compared with control; #*p* < 0.05, ##*p* < 0.01, ###*p* < 0.001 compared with inflammatory stimulants. Con: controls. IL: interleukin. Hc: hydrocortisone. LPS: lipopolysaccharide. TNF-α: tumor necrosis factor-alpha. IFN-γ: interferon-gamma.

Reverse transcription-polymerase chain reaction (RT-PCR) was used to examine the influence of LPS, TNF-α, and IFN-γ as inflammatory stimulants on the mRNA expression levels of pro-inflammatory cytokines in the presence and absence of ASex. LPS treatment increased the expression levels of all inflammatory cytokines in HDFs (Figure 5). The IL-1β level was increased by 7.8-fold, which was suppressed by ASex, and was reduced by 1.6-fold with treatment of hydrocortisone (Hc) as a positive control. The IL-6 level increased by 3.4-fold, was almost completely suppressed by ASex, and reduced by 1.2-fold by Hc. IL-8 and IL-10 levels increased by 1.2-fold and were almost completely suppressed by ASex. The IL-18 level was increased by 1.2-fold and was almost completely suppressed by high concentrations of both ASex and Hc. IL-33 and TNF-α levels were increased by 2-fold and 8.9-fold, respectively, and these increases were completely inhibited by ASex.

Treatment of TNF-α also increased the mRNA expression levels of inflammatory cytokines, except for IL-18, which is a representative chronic inflammatory cytokine. IL-6 and IL-10 levels were increased by 3- and 1.1-fold, respectively, and the other cytokines were increased by more than 5-fold. Treatment with ASex completely decreased the increased expression of all cytokines, except for IL-18.

Since the chronic phase of inflammation cannot be induced in a cell model, we treated the HDFs with IFN-γ, which is produced at the chronic stage of inflammation, to mimic chronic skin inflammation. IFN-γ treatment induced no significant change in the mRNA levels of IL-1β and IL-8. However, the levels of other cytokines (IL-6, IL-10, and TNF-α) increased by approximately 1.5-fold, and the IL-33 level increased by 2.2-fold. ASex treatment almost completely suppressed these increased expression levels for all cytokines.

### 2.3. In Vivo Model

#### 2.3.1. Clinical Observations

**Figure 6:**
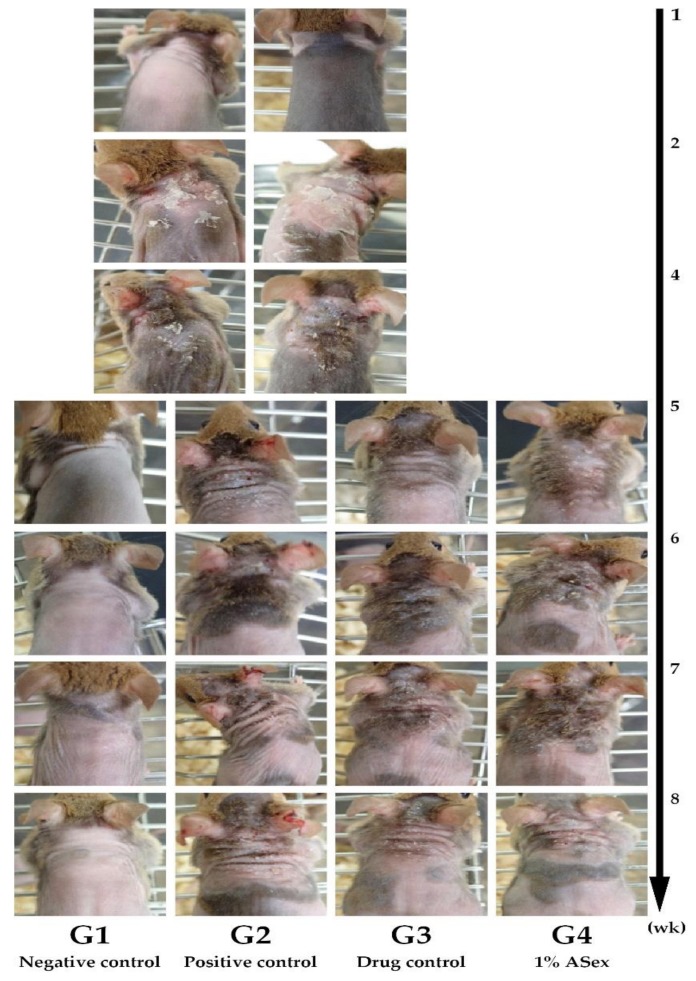
Clinical observations in NC/Nga mice. HDM: house dust mite ointment. G1: negative control; G2: positive control (HDM only); G3: drug control (0.1% Hc); G4: 1% ASex. All groups were induced by HDM except for G1.

Skin lesions were induced in NC/Nga mice by repeated treatment with HDM, a known major allergen that causes skin inflammation. We analyzed the 8-week course of clinical and immunological changes, including the onset and progression of the condition (Figure 1b). At week 5, skin inflammation signs such as dryness, erythema, and edema were observed, which are typical of the acute phases of human AD. Over time, the skin inflammation gradually recovered and features of the sub-chronic phase began to appear (Figure 6).

#### 2.3.2. Histological Evaluation

**Figure 7:**
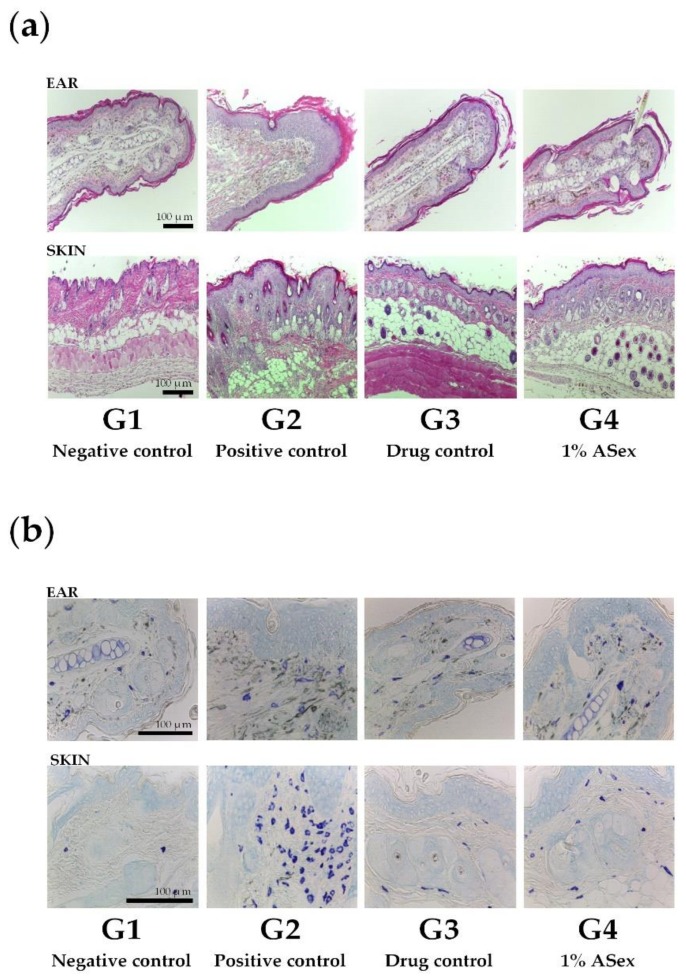
Histological analysis of NC/Nga mice. (**a**) Hematoxylin and eosin staining; (**b**) Toluene blue staining. G1: negative control group; G2: positive control group (HDM only); G3: drug control (0.1% Hc); G4: 1% ASex. All groups were induced with house dust mite ointment except for G1.

Hematoxylin and eosin (H&E) staining of the ears and dorsal skin tissue of HDM-treated NC/Nga mice (G2) showed significantly increased epidermal thickening and lymphocytes infiltration compared with the negative control group, G1 (Figure 7a). In addition, we observed similar clinical phenotypes to human AD, including epidermal hyperplasia, epidermal spongiosis, epidermal acanthosis, and epidermal elongation of the rete ridges. Epidermal and dermal thickening along with the lesions were significantly reduced, and the lymphocyte infiltration was significantly decreased in G3 (treated with Hc) and G4 (treated with 1% ASex). These results showed that ASex could restore the epidermis as effectively as Hc.

Toluene blue staining of the ears and dorsal skin tissue in HDM-treated NC/Nga mice showed a relatively higher number of infiltrated mast cells in G2 than in G1. However, compared with G2, the number of infiltrated mast cells was significantly reduced in G3 and G4, with similar effects for Hc and ASex (Figure 7b).

#### 2.3.3. Scratching Behavior Test

**Figure 8:**
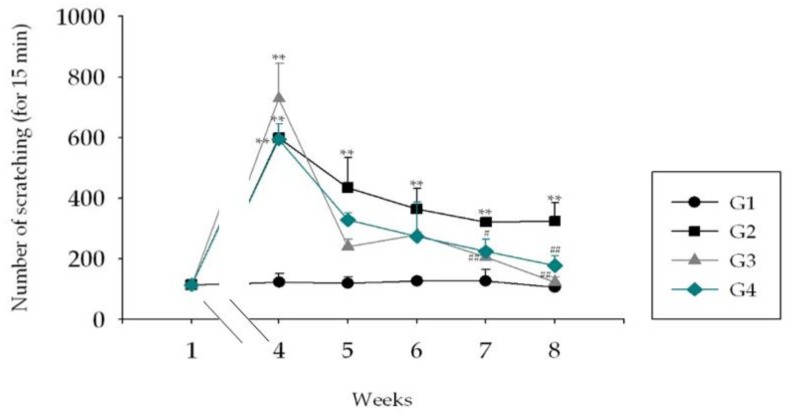
Scratching behavior in NC/Nga mice. G1: negative control group; G2: positive control group (HDM only); G3: drug control (0.1% Hc); G4: 1% ASex. All groups were induced with HDM except for G1. The results are expressed as the mean ± SD of triplicate experiments (*n* = 3). Statistical significance was assessed by ANOVA: *p < 0.05, **p < 0.01 compared with G1. #*p* < 0.05, ##*p* < 0.01 compared with G2.

In all HDM-treated NC/Nga mouse groups, the number of scratches of the skin lesions increased by approximately 600 times compared to that of G1 mice at week 4 (Figure 8). This behavior remained increased by about 400 times in G2 until the end of week 8. However, treatment with Hc (G3) and 1% ASex (G4) in HDM-induced NC/Nga mice significantly reduced the numbers of scratches.

#### 2.3.4. Serum IgE and Cytokines

**Figure 9:**
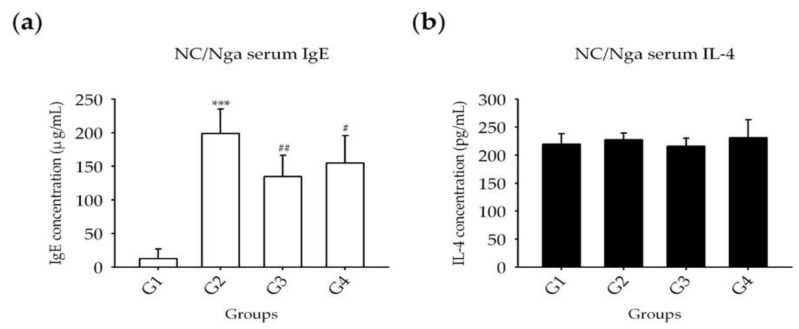

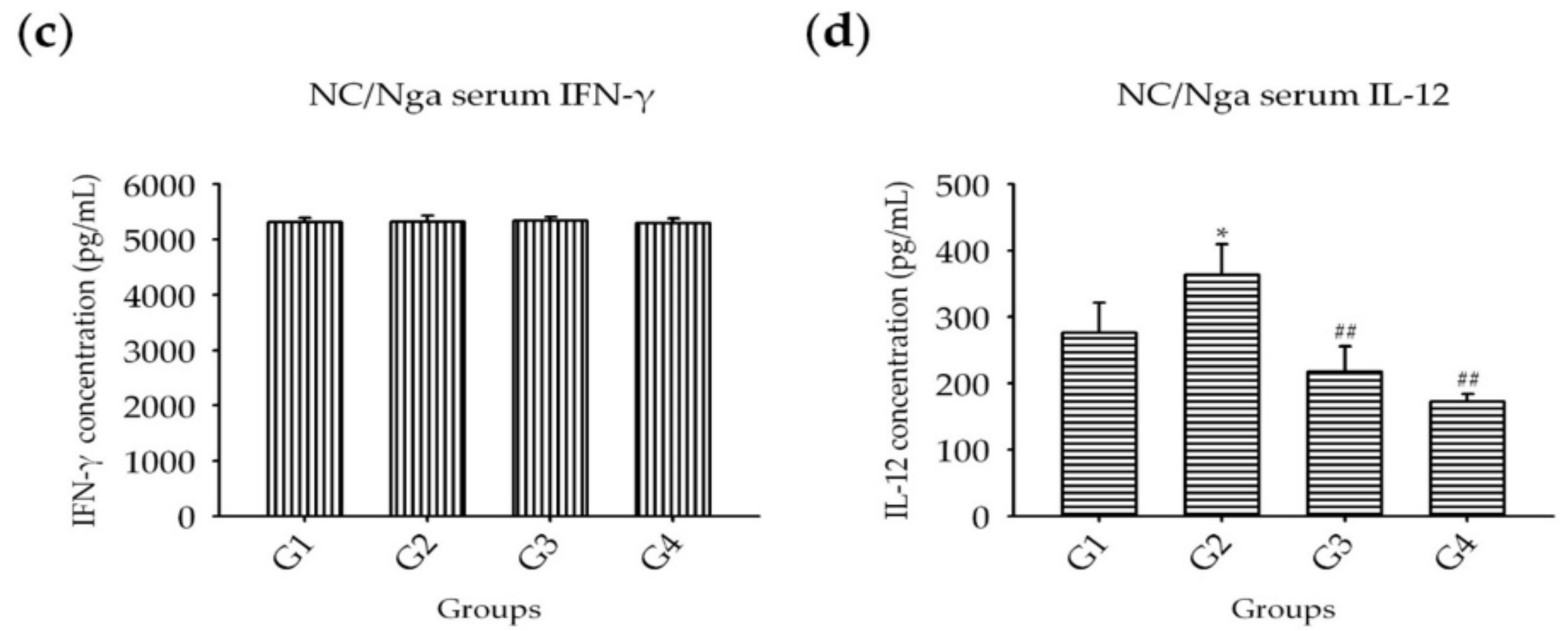
Serum IgE and cytokine levels after ASex treatment in NC/Nga mice. (**a**) Level of serum IgE; (**b**) level of IL-4; (**c**) level of IFN-γ; (**d**) level of IL-12. G1: negative control group; G2: positive control group (HDM only); G3: drug control (0.1% Hc); G4: 1% ASex. All groups were induced with HDM except for G1. The results are expressed as the mean ± SD of triplicate experiments (*n* = 3). Statistical significance was assessed by ANOVA: *p < 0.05, ***p < 0.001 compared with G1. #*p* < 0.05, ##*p* < 0.01 compared with G2.

Blood samples were collected at sacrifice, and serum IgE, IL-4, IL-12, and IFN-γ levels were analyzed by enzyme-linked immunoassay (ELISA). Similar changes were observed in the levels of IgE and cytokines. Compared with G1, the IgE level was significantly increased in all groups (G2–G4); In G3 and G4, showed a significantly decrease compared with G2 (Figure 9a). IL-4 and IFN-γ levels were not significantly different among all groups (Figure 9b,c). IL-12 levels were significantly increased in G2 compared with those of G1 and exhibited a greater decrease in G4 than in G3 compared with G2 (Figure 9d).

#### 2.3.5. Expression of Inflammatory Cytokines Induced by HDM in NC/Nga Mice

**Figure 10:**
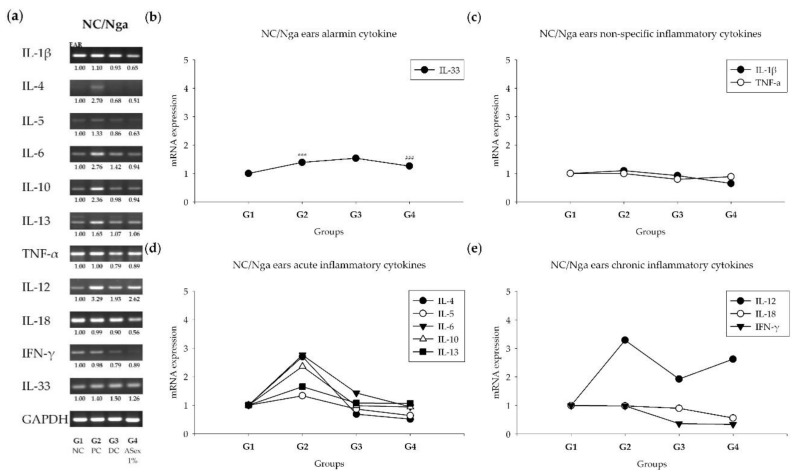
Expression of inflammatory cytokines in the ears of NC/Nga mice. (**a**): All of the inflammatory cytokines mRNA expression in ears of NC/Nga mice; (**b**): Level of alarmin cytokine; (**c**): level of non-specific inflammatory cytokines; (**d**): level of acute inflammatory cytokines; (**e**): level of chronic inflammatory cytokines. G1: negative control group; G2: positive control group (HDM only); G3: drug control (0.1% Hc); G4: 1% ASex. All groups were induced with HDM except for G1. The results are expressed as the mean ± SD of triplicate experiments (*n* = 3). Statistical significance was assessed by Student’s *t*-test and ANOVA: *p < 0.05, ***p < 0.001 compared with G1; ##*p* < 0.01, ###*p* < 0.001 compared with G2.

RT-PCR of the ear tissues showed that the mRNA levels of IL-1β and IL-5 were increased by approximately 1.2-fold; IL-4, IL-6, IL-10, and IL-13 levels increased by approximately 2-fold; IL-12 increased by approximately 3.2-fold; and IL-33 increased by 1.4-fold in G2 compared with those of G1 (Figure 10). These increases were significantly inhibited by Hc (G3), except for that of IL-33. ASex (G4) showed similar inhibitory effects on cytokine gene expression, and the HDM-induced expression of IL-4, IL-5, IL-6, and IL-33 was more effectively inhibited in G4 than in G3. No changes in the levels of TNF-α, IL-18, and IFN-γ were detected, but the normal level expression of IL-18 was also inhibited in G4.

Changes in cytokines in the skin of the HDM-treated NC/Nga mice were less obvious than those in the ears. In the skin, IL-1β and IL-33 levels were increased by approximately 1.2-fold in G2 compared with those of G1. IL-1β levels were slightly decreased in G3 and were decreased by 0.6-fold in G4; however, the increase in IL-33 levels was not inhibited in G3 and G4. Thus, in G2, cytokines released in the chronic phase were suppressed to below normal levels, and other cytokines showed no significant changes (data not shown).

#### 2.3.6. Expression of Inflammatory Cytokines

**Figure 11:**
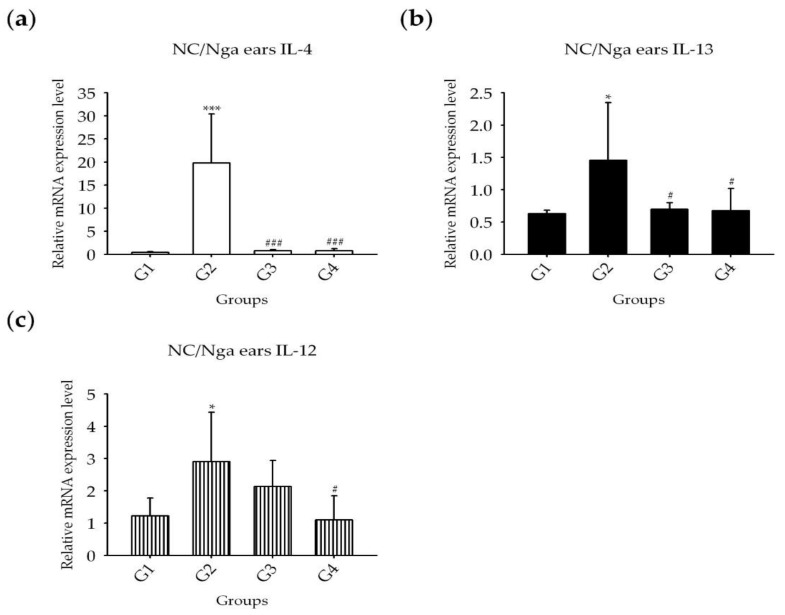
Expression of inflammatory cytokines in the ears of NC/Nga mice analyzed by RT-qPCR. (**a**): Level of IL-4; (**b**): level of IL-13; (**c**): level of IL-12. G: negative control group; G2: positive control group (HDM only); G3: drug control (0.1% Hc); G4: 1% ASex. All groups were induced with HDM except for G1. The results are expressed as the mean ± SD of triplicate experiments (*n* = 3). Statistical significance was assessed by ANOVA: *p < 0.05, ***p < 0.001 compared with G1; #*p* < 0.05, ###*p* < 0.001 compared with G2.

Quantitative RT-PCR (RT-qPCR) showed that the relative quantification of mRNA expression levels of the Th2 cytokines IL-4 and IL-13, and the Th1 cytokine IL-12 in the ear tissues were significantly increased in G2 compared with those of G1 (Figure 11). The increases in IL-4 and IL-12 were significantly suppressed in G3 and G4 compared with G2, and the increase in IL-12 was significantly suppressed in G4 compared to G2.

## 3. Discussion

The complex etiology of skin inflammation makes it difficult to define objective indicators of diagnosis and prognosis. Therefore, it is crucial to establish an experimental model of appropriate skin inflammation and evaluate the similarities and differences compared to humans. However, the precise criteria for determining the occurrence and progression of skin inflammation (acute to chronic phase) in human and animal models remain controversial. To help resolve this issue, we examined the clinical and/or immunological aspects of skin inflammation progression/exacerbation using an in vitro model of human fibroblasts for observing inflammation in the acute phase and an in vivo model of NC/Nga mice for observing inflammation in the sub-chronic/chronic phase. We further used these models to evaluate the applicability of ASex as an anti-inflammatory agent. ASex contains several components, including diarlyheptanoids, such as oregonin and hirsutenone, as the major components. Choi et al. [3] reported the anti-inflammatory effects of whole extracts of *A.* *sibirica* collected from various regions of Korea rather than a single component using an in vitro model. However, the *A.* *sibirica* bark of the Jacheon area could be easily collected in large quantities and the contents of indicator materials were much higher than those of other areas. Therefore, we focused on ASex from samples collected in the Jacheon area using our in vitro and in vivo models.

The LDH assay demonstrated no toxicity of ASex to HDFs. We also confirmed the lack of a cell death effect of ASex using the MTT (3-(4,5-Dimethylthiazol-2-yl)-2,5-diphenyltetrazolium bromide) assay (data not shown), which actually indicated that treatment with ASex for 3 h and 12 h increased cell proliferation. However, direct counting of cell numbers did not confirm this effect. This false-positive result might be related to the reaction of the major components of the overall plant extract (e.g. flavonols, kaempferol) with MTT to immediately form formazan as the detection product [26]. The ASex used in our experiment also contains high amounts of flavonol. Therefore, the increased cell proliferation pattern was likely due to the reaction of the MTT reagent with the flavonoids of ASex, indicating that ASex might not promote cell proliferation directly.

The in vitro model was established to represent skin inflammation in the acute phase. Indeed, after the treatment of HDFs with inflammatory stimulants (LPS, TNF-α, and IFN-γ), the levels of acute inflammatory cytokines (IL-6, IL-10), an alarmin cytokine (IL-33), and non-specific cytokines (IL-1β, IL-8) increased. Although the level of IL-18 was barely affected by the stimulants, it was increased by both Hc and a low concentration of ASex. As IL-18 expression is a marker of chronic inflammation, this result reflects a balance through the negative feedback loop of Th1/2 cytokines [4]. In other words, since Th2 cytokine expression is strongly inhibited by Hc and low ASex concentrations, the Th1/2 balance may be tipped toward Th1, resulting in increased IL-18 expression. In contrast, a high concentration of ASex inhibited the expression of both Th1 and Th2 cytokines.

Choi et al. [3] suggested that HDFs may be involved in the positive feedback loop of the Th1 and Th2 balance by increasing IL-33 levels under IFN-γ treatment. We also found that IL-33 levels increased under IFN-γ stimulation, and this change was strongly inhibited by ASex. These results suggested that ASex can block a sustained inflammatory response through inhibition of the positive feedback loop of acute and chronic inflammation. When IL-33 initiates inflammation, Th2 cytokines involved in acute inflammation are secreted, and repetition/aggravation of acute skin inflammation results in secretion of Th1 cytokines involved in chronic inflammation. Thus, Th2 and Th1 cytokines maintain a negative feedback balance. However, the cytokine IFN-γ, which has a profound effect on chronic inflammation progression, can be directly stimulated by IL-33 as an inflammation initiation factor, without requiring a repetition/aggravation cycle of acute inflammation. Crucially, ASex is able to generally suppress these markers of inflammation, indicating that it could prevent this cycle of aggravation. In addition, considering the characteristics and chemical structure of the ASex components, it would be possible to hypothesize that the mechanisms linked to the protective effects might be mediated by an increase in antioxidant activity, via activation of the transcription factor Nrf2 (Nuclear factor erythroid 2-related factor). This factor is modulated by molecules that have antioxidant characteristics [27]. The modulation of the antioxidant response is directly related to the inflammatory response because the activation of NF-kB is redox-sensitive. Therefore, the potential activation of Nrf2 would generate an inactivation of NF-kB [28].

NC/Nga mice exhibit increased serum IgE production and skin inflammation in response to allergens. However, the frequency and severity of skin lesions may vary in normal environments, and repeated exposure to specific stimuli is necessary to induce inflammatory skin lesions. Repeated induction of HDM promotes the secretion of acute- and chronic-phase cytokines involved in skin inflammation, intensifies the inflammation, and induces AD-like skin lesions through IgE hyper-production [24,25,29]. Therefore, we chose this in vivo model to represent skin inflammation in the acute and sub-chronic phase. We expected this model to eventually enter the chronic phase; however, this was not possible with our 8-week protocol. Thus, a lesion similar to the developmental stage of an AD-like skin lesion was observed, although this was not a typical AD-like skin lesion. This difference was apparent in the induction and observation of skin lesions as the epidermal layer of the ear is thinner, the dermis and the subcutaneous fat layer are thinner, and there is less fat than on the dorsal skin. Epidermal thickening and increased infiltration of inflammatory cells in stained skin sections showed the typical histological characteristics of skin inflammation. Serum IgE and IL-12 levels increased significantly. Acute-phase cytokines (IL-4, IL-5, IL-6, IL-10, and IL-13), a sub-chronic-phase cytokine (IL-12), and an alarmin cytokine (IL-33) typically exhibited greater increases in the ears than in the skin lesions. The scratching behavior test also demonstrated increased itching, an essential symptom of skin inflammation. As shown by Hashimoto [30], scratching behaviors increased significantly with the severity of skin inflammation. In our study, the HDM-treated NC/Nga mice exhibited significantly increased scratching compared to the negative control group, and this response was alleviated by treatment with Hc and ASex. These results indicated that ASex had an anti-inflammatory effect similar to Hc, and inhibited the histological and serological changes associated with inflammation.

ASex exerts clear anti-inflammatory effects through the suppression of immunological changes in the serum and tissues, resulting in the amelioration of the histological changes in skin lesions. Thus, we suggest that ASex is a promising anti-inflammatory candidate that can act effectively inhibit the production of Th1/2 cytokines and the positive feedback Th1/2 loop to restore the histopathological changes that occur during inflammation.

## 4. Materials and Methods

### 4.1. Preparation of ASex

#### 4.1.1. Plant Material Extraction and Isolation

We used the extracts from the barks of *Alnus sibirica* in Jacheon, Korea. The identification of the components was certified by Prof. Minwon Lee (College of Pharmacognosy, Chung-Ang University, Seoul, Korea), and a voucher specimen was deposited at the pharmacy department’s herbarium. The bark (450 g) was extracted three times with 70% proethanol A (Cat. No. J5V108, Reagents DUKSAN, Gyeonggi, Korea) at 70 °C under reflux. After the removal of proethanol A and ethyl acetate under vacuum, the total yield was 48.28 g.

#### 4.1.2. HPLC

HPLC (Waters 600 system, Marshall Scientific, Hampton, NH, USA) and a Hector C^18^ column (5 μm, 4.6 × 250 mm) were used for quantitative analysis of the compounds in ASex. The mobile phase consisted of solvents A (H_2_O) and B (acetonitrile), which were described and filtered by Whatman membrane filters (0.2 μm, diameter 47 mm) (Table 2). The sample solution was 5 mg of ASex from Jacheon, Geochang, and Chungnam, dissolved in 1 mL of 70% MeOH. The standard solution was prepared by taking 2 mg of oregonin and hirsutenone, respectively, which were dissolved in 70% MeOH to prepare a stock solution, and diluted. Oregonin concentrations of 2000, 1000, 500, and 250 μg/mL, and hirsutenone concentrations of 1000, 500, 250, 125, and 62.5 μg/mL were prepared.

### 4.2. In Vitro Models

#### 4.2.1. Preparation of ASex and Inflammatory Stimulants

ASex was dissolved in 70% ethanol. The ASex used in the experiments were 100,000 and 500,000 μg/mL stock solutions and diluted to the required concentration according to the final volume. Hc, LPS, TNF-α, and IFN-γ were purchased from Sigma-Aldrich (St. Louis, MO, USA); 100 μg/mL stock solutions were prepared and diluted to the required concentration according to the final volume as follows: 100 μg/mL Hc (for the same as ASex), 100 ng/mL LPS (for 3 h treatment), 10 ng/mL TNF-α (for 12 h treatment), and 10 ng/mL IFN-γ (for 12 h treatment) (Figure 1a).

#### 4.2.2. Cell Culture

Primary HDFs (Cat. No. C-013-5C) were cultured in Medium 106 (Cat. No. M106-500) containing low serum growth supplement kits (LSGS kit, Cat. No. S-003-K); the cells, medium, and supplement kits were purchased from Invitrogen Life Technologies Inc. (GIBCO Cascade Biologics, Waltham, MA, USA). The cells were maintained in the culture plate at 37 °C in a humidified atmosphere with 5% CO_2_. For all experiments, the cells were grown to greater than 80% confluence and subjected to no more than 15 passages.

#### 4.2.3. Cytotoxicity: LDH assay

The LDH cytotoxicity assay kit (Cat. No. 88954, Pierce, Rockford, IL, USA) was used to determine the cytotoxicity of compounds. HDFs were seeded at approximately 5 × 10^3^ cells/well in each well of a 96-well plate and incubated overnight in 5% CO_2_ at 37 °C. The medium containing 100, 300, and 1000 μg/mL ASex was added, and the cells were further incubated for 3 h and 12 h. The medium was removed, and the working solution was added in accordance with the manufacturer’s instructions. LDH activity was proportional to the rate of pyruvate loss, which was assayed by the absorbance change using a microplate reader (Sunrise, Tecan, Männedorf, Zürich, Switzerland).

#### 4.2.4. Expression of Inflammatory Cytokines Induced by Inflammatory Stimulants in HDFs: RT-PCR

HDFs were seeded at a density of 5 × 10^5^ cells per 10-mm culture plate in 10 mL medium. The cells were incubated at 37 °C in 5% CO_2_ overnight and allowed to adhere. The medium was changed to starvation medium for 1 h and the cells were treated with 300 and 1000 μg/mL ASex for 1 h before treatment with the inflammatory stimulants as follows: 100 ng/mL LPS for 3 h, or 10 ng/mL TNF-α and 10 ng/mL IFN-γ for 12 h. The methods for RNA isolation and RT-PCR analysis followed those described by Choi et al. [3]. All primers used in the RT-PCR analysis are presented in Table 3. We designed the primer sequences on the basis of known mRNA sequences (Table 2) by using Entrez (NCBI, NIH, Bethesda, MD, USA). The mRNA levels were normalized with the density of GAPDH in the control group.

### 4.3. In Vivo Model

#### 4.3.1. Animals

Seven-week-old female specific pathogen-free NC/Nga mice were purchased from Central Laboratories (Japan SLC, Shizuoka, Japan). The animals were maintained in conventional conditions for 2 weeks before the experiments were started [31]. All animal experiments were performed in accordance with the regulations and with approval from the Institutional Animal Care and Use Committee (IACUC) of Chung-Ang University (Approval No. 201800012). The animals were randomized to the control (G1, no treatment) positive control (G2, HDM only), drug control (G3, 0.1% Hc), and 1% ASex group (G4) with six mice per group.

#### 4.3.2. HDM and ASex Treatment

To apply HDM, the fur was shaved from the back and behind the ears of the mice using clippers and a shaver. We also used a depilatory cream to remove the hair thoroughly only at the first induction. From the second induction, we shaved the hair on the back and the ears before treatment using clippers, and then spread 150 μL of 4% sodium dodecyl sulfate solution (Sigma-Aldrich, St. Louis, MO, USA) evenly on the shaved dorsal skin and both surfaces of each ear to disrupt the skin barrier. Skin inflammation was induced according to the methods described by Yamamoto et al. [25]; 100 mg HDM (*Dermatophagoides farinae* extracts; Biostir Ins., Osaka, Japan) was applied on the back and both surfaces of ears uniformly twice per week for 8 weeks. The 1% (*w*/*w*) ASex cream and 0.1% (*w*/*w*) Hc cream (provided by Prof. Young Wook Choi, College of Pharmacy, Chung-Ang University, Seoul, Korea) was applied topically to the lesions from Monday to Friday for 4 weeks. After the end of week 8, all mice were sacrificed.

#### 4.3.3. Histological Evaluation

The skin removed from the lesion site was fixed in 4% (*w*/*v*) neutral phosphate-buffered formalin (pH 7.2) for 2 h at room temperature (25 °C), embedded in paraffin, and serially sectioned to a thickness of 4 μm. The slices were stained with H&E to assess epidermal hyperplasia and with toluene blue to assess infiltration of cells.

#### 4.3.4. Scratching Behavior Test

Video recordings of scratching behavior were obtained twice per week for week 1 and then from week 5 to 8 consecutively. The number of times a mouse scratched the ears and dorsal skin lesions for 15 min was counted (not counting grooming or scratching other parts of the body). The scratching behavior was calculated from the mean value of each group.

#### 4.3.5. Serum IgE and Cytokines Measurement

After the mice were sacrificed and serum was obtained, total serum IgE, IL-4, IL-12, and IFN-γ levels were measured using the respective mouse ELISA kit (Invitrogen, Waltham, MA, USA) in accordance with the manufacturer’s instructions.

#### 4.3.6. Expression of Inflammatory Cytokines Induced by HDM in NC/Nga Mice

RT-PCR was performed as described for the in vitro analysis from the tissues collected from mice in each group.

### 4.4. Statistical Analysis

All data are expressed as the mean ± SD. All analyses were performed from six independent experiments in which three replicate analyses were conducted. Statistical significance was assessed by Student’s *t*-tests to compare means between two groups, or with one-way or two-way analysis of variance for multiple comparisons followed by Tukey HSD or Dunnett T3 post-hoc tests. The analyses were performed using the SPSS version 25 KO software package (IBM SPSS, Chicago, IL, USA).

## 5. Conclusions

In this study, we evaluated the anti-inflammation effects of ASex on in vitro and in vivo models. We examined the clinical and immunological aspects of skin inflammation using human fibroblasts as an in vitro model of acute phase inflammation and house dust mite-exposed NC/Nga mice as an in vivo model of the sub-chronic/chronic phase inflammation. In these models showed progression and exacerbation of skin inflammation based on elevation of alarmin cytokines, non-specific/acute/chronic inflammatory cytokines induced by inflammatory stimulants.

The ASex restored to the histopathological changes that occur during skin inflammation, and showed similar or superior anti-inflammatory effects to Hc. Therefore, it is a promising anti-inflammatory candidate that can act effectively inhibit the production of Th1/2 cytokines and reduced side effects than Hc. The factors for occurrence/progress of skin inflammation (acute to chronic phase) remain unclear owing to their complexity, which limits diagnosis and development of effective, targeted drugs for treating skin inflammation conditions such as atopic dermatitis. Therefore, we would providing to effective models for alleviating the complex network of chemokine/cytokine cascades involved in skin inflammation, on testing new compounds including natural products.

## Figures and Tables

**Table 1:** ASex yield (%) and indicator content (%) from each region determined by high-performance liquid chromatography (HPLC).

Region	Part	ASex Yield Relative to AS	Oregonin	Hirsutenone
Jacheon, Korea	Leaves	14.8	2.97	0.49
Jacheon, Korea	Barks	6.91	12.26	0.71
Geochang, Korea	Leaves	22.2	1.35	0.40
Geochang, Korea	Barks	8.7	8.85	0.74
Chuncheon, Korea	Leaves	11.69	2.58	0.51
Chuncheon, Korea	Barks	8.7	9.70	0.82

ASex: *Alnus sibirica* extract. AS: *Alnus sibirica*. %: percentage.

**Table 2:** Analysis condition of high-performance liquid chromatography.

Column	Hector C^18^ HPLC column (5 µm, 250 × 4.6 mm)						
Detector	Waters 112 UV/V_IS_ (280 nm)						
	0 min	20 min	35 min	38 min	45 min	47 min	55 min
H_2_O	95	75	60	0	0	95	95
Acetonitrile	5	25	40	100	100	5	5

**Table 3:** Primer sequences used in PCR.

Primer	bp	°C	Sense	Antisense
uGAPDH	343	58	5′-CAGTGAGCTTCCCGTTCAG-3′	5′-GCCAAAAGGGTCATCATCTC-3′
hIL-1β	391	62	5′-AAACAGATGAAGTGCTCCTTCCAGG-3	5′-TGGAGAACACCACTTGTTGCTCCA-3′
hIL-6	264	62	5′-ATGAACTCCTTCTCCACAAGC-3′	5′-GTTTTCTGCCAGTGCCTCTTTG-3′
hIL-8	293	58	5′-ATGACTTCCAAGCTGGCCGTGGCT-3′	5′-TCTCAGCCCTCTTCAAAAACTTCTC-3′
hIL-10	352	55	5′-CTGAGAACCAAGACCCAGACATCAAGG-3′	5′-CAATAAGGTTTCTCAAGGGGCTGG-3′
hIL-18	342	53	5′-GCTTGAATCTAAATTATCAGTC-3′	5′-GAAGATTCAAATTGCATCTTAT-3′
hIL-33	180	58	5′-CAAAGAAGTTTGCCCCATGT-3′	5′-AAGGCAAAGCACTCCACAGT-3′
hTNF-α	237	62	5′-GAGCTGAGAGATAACCAGCTGGTG-3′	5′-CAGATAGATGGGCTCATACCAGGG-3′
mIL-1β	213	53	5’-AAGGAGAACCAAGCAACGACAAAA-3’	5’-TGGGGAACTCTGCAGACTCAAACT-3’
mIL-4	95	62	5′-ACAGGAGAAGGGACGCCAT-3′	5′-GAAGCCCTACAGACGAGCTCA-3′
mIL-5	277	54	5′-GAAAGAGACCTTGACACAGCT-3′	5′-GAACTCTTGCAGGTAATCCAG-3′
mIL-6	141	62	5′-AGGATACCACTCCCAACAGACCT-3′	5′-CAAGTGCATCATCGTTGTTCATAC-3′
mIL-10	252	55	5′-TACCTGGTAGAAGTGATGCC-3′	5′-CATCATGTATGCTTCTATGC-3′
mIL-12	178	62	5′-GGAAGCAC GCAGCAGAATAA-3′	5′-CTTGAGGGAGAAGTAGGAATG-3′
mIL-13	220	58	5′-TCTTGCTTGCCTTGGTGGTCTCGC-3′	5′-GATGGCATTGCAATTGGAGAT-3′
mIL-18	434	53	5′-ACTGTACAACCGCAGTAATACGC-3′	5′-AGTGAACATTACAGATTTATCCC-3′
mIL-33	155	60	5′-GGTGTGGATGGGAAGAAGCTG -3′	5′-GAGGACTTTTTGTGAAGGACG -3′
mTNF-α	384	58	5′-GCGACGTGGAACTGGCAGAAG-3′	5′-GGTACAACCCATCGGCTGGCA-3′
mIFN-γ	252	60	5′-AACTCAAGTGGCATAGATGTGG-3′	5′-GACCTCAAACTTGGCAATACTCATGA-3′

bp: base pair of product sizes. °C: annealing temperature. u: universal. h: human. m: mouse. GAPDH: glyceraldehyde 3-phosphate dehydrogenase. IL: interleukin.

## Abbreviations

MDPI	Multidisciplinary Digital Publishing Institute
DOAJ	Directory of open access journals
TLA	Three letter acronym
LD	linear dichroism
